# Enhancing Africa’s agriculture and food systems through responsible and gender inclusive AI innovation: insights from AI4AFS network

**DOI:** 10.3389/frai.2024.1472236

**Published:** 2025-01-23

**Authors:** Nicholas Ozor, Joel Nwakaire, Alfred Nyambane, Wentland Muhatiah, Cynthia Nwobodo

**Affiliations:** African Technology Policy Studies Network (ATPS), Nairobi, Kenya

**Keywords:** responsible AI, gender equality and social inclusion, food security, sustainable agriculture and food system, capacity building, co-creation

## Abstract

The integration of artificial intelligence (AI) technologies into agriculture holds urgent and transformative potential for enhancing food security across Sub-Saharan Africa (SSA), a region acutely impacted by climate change and resource constraints. This paper examines experiences from the Artificial Intelligence for Agriculture and Food Systems (AI4AFS) Innovation Research Network, which provided funding to innovative projects in eight SSA countries. Through a set of case studies, we explore AI-driven solutions for pest and disease detection across crops such as cashew, maize, tomato, and cassava, including a real-time health monitoring tool for Nsukka Yellow pepper. Using participatory design, and key informant interview, robust monitoring and evaluation, and incorporating ethical frameworks, the research prioritizes gender equality, social inclusion, and environmental sustainability in AI development and deployment. Our results demonstrate that responsible AI practices can significantly enhance agricultural productivity while maintaining low carbon footprints. This research offers a unique, localized perspective on AI’s role in addressing SSA’s agricultural challenges, with implications for global food security as demand rises and environmental resources shrink. Key recommendations include establishing robust policy frameworks, strengthening capacity-building efforts, and securing sustainable funding mechanisms to support long-term AI adoption. This work provides the global community, policymakers, and stakeholders with critical insights on establishing ethical, responsible, and inclusive AI practices that can be adapted to similar agricultural contexts worldwide, contributing to sustainable food systems on an international scale.

## 1 Introduction

### 1.1 Background and overview of the artificial intelligence for agriculture and food systems innovation research network

Africa’s agriculture and food systems face significant challenges due to climate change. The continent is highly vulnerable to climate impacts, with Sub-Saharan Africa being particularly prone to droughts and extreme weather events (Husen, 2023). The changing climate patterns, including intense drought, erratic rainfall and floods, pose a threat to food security by impacting crop production and food availability (Nkwi, 2023). Despite efforts to enhance agricultural productivity through irrigation expansion, a 3°C warmer climate could lead to a significant food deficit, affecting over half of the projected population of 2.6 billion by 2050 (Muleta et al., 2023). Addressing climate change impacts on agriculture is crucial to ensure food security and mitigate the risks posed by climate variability in Africa. This will require an increase in agricultural and food production by up to 70% to fit the need of the population, a serious challenge for the agriculture and food systems. Also the rising fossil fuel prices are accelerating the shift towards biofuel production, as countries seek affordable, renewable energy alternatives to costly traditional fuels (Vochozka et al., 2020a,b). The restriction of peoples’ movement during the COVID-19 pandemic played a vital role in global air quality concentration levels, reducing emissions according to Manigandan Sekar et al. (2021). The demand for biofuels places higher demand on agricultural production, especially in crops like corn, soybean, and sugarcane, which are primary biofuel sources. This increased agricultural focus aims to reduce dependency on oil, stabilize fuel prices, and improve energy security. However, the growth in biofuel production also raises concerns over food supply and land use, as more land and resources are allocated to energy crops rather than food production, impacting global food markets. Such requirements, in a context of resource scarcity, climate change, COVID-19 pandemic, increase demand on biofuels, and very harsh socioeconomic conjecture, is difficult to attain without the intervention of emerging technologies and innovations such as artificial intelligence to leapfrog the transformations required in the sector. The impacts of climate change on agriculture are a global concern, affecting not only Sub-Saharan Africa but also regions with similar vulnerabilities. Addressing these impacts aligns with global sustainability strategies, offering insights applicable beyond Africa (Nkwi, 2023; Muleta et al., 2023). For instance, artificial intelligence (AI) has shown promising results in agricultural fruit prediction by improving accuracy through deep neural networks and econometric models. These AI-driven methods have been effective in forecasting agricultural output, as seen in studies that employed deep learning and machine learning techniques to predict fruit production (Khan et al., 2020; Rehman et al., 2018). AI-based predictive models address complex economic challenges, supporting decision-making in agricultural production, market dynamics, and resilience strategies (NHS England, 2019). Artificial intelligence has been applied in predicting oil prices using promissing type of artificial neural network-LSTM (Long Short-Term Memory) (Vochozka et al., 2020a,b). More so, Anaba et al. (2024) explored the potential benefits and implications of incorporating AI into maintenance strategies within industrial environments, contributing to increased efficiency. Kliestik et al. (2023) affirms the usefulness of AI in predictive maintenance of agricultural processing machinery, thus enhancing maintenance practices in industrial settings which ensure continuous food process, thus contributing sustained food availability and stability. These advancements could play a vital role in addressing food security challenges by enhancing agricultural productivity and resilience in Africa’s climate-vulnerable regions.

The Artificial Intelligence for Agriculture and Food Systems Innovation Research Network (AI4AFS) is part of the innovation stream of the Artificial Intelligence for Development Africa (AI4D Africa) program dedicated to a future where Africans across all regions create and use artificial intelligence to lead healthier, happier, and greener lives. The AI4AFS program aims to deliver at least one AI-based solutions per country where the grantees are based, engage at least 100 farmers per project, and demonstrate a minimum 20% improvement in crop yield or disease detection outcomes. Achieving sustainable and adequate food supply to meet the demands of this teeming population requires the adoption of emerging technologies and the responsible AI technologies stand out among many to accomplish this daunting task. The general goal of the innovation research network is *to advance the responsible development, deployment and scaling of home-grown AI innovations to tackle pressing challenges in agriculture and food systems in Africa*. Achieving this objective would make a significant contribution to agriculture, food and nutritional security as well as enhance the livelihoods of African people especially women and youth who form the majority of the small-scale farmers in Africa. By developing, deploying and scaling AI innovations that are home-grown, peculiar agricultural production and productivity challenges unique to Africa can be addressed. Overall, there will be adequate endogenous capacity, technology and innovations, infrastructure, and enabling policy environment to tackle pressing AFS challenges to achieve sustainable transformation in the sector and overall economies on the continent.

Three sets of interventions were implemented by the partners in the Hub to achieve the stated objective. These include setting up an artificial intelligence innovation research network for agriculture and food systems; managing the innovation research network; and fostering collaborations, knowledge exchange and valorization among the network and beyond. The Network understands that to promote wide-spread adoption of AI, there is need to involve or collaborate with Small and Medium-sized enterprises (SMEs) in the development of AI tools according to Dvorský et al. (2023). SMEs play a significant role in countries economies as they are the engine of growth, especially in developing and emerging economies. Established in the fall of 2021, the program has provided sub-grants to innovative projects in eight countries namely; Ghana, Kenya, Malawi, Nigeria, Senegal, Tanzania, and Uganda, fostering the development and deployment of responsible AI solutions that prioritize equity, fairness, ethical principles, and gender equality. It is expected that through these interventions, responsible and homegrown artificial intelligence research and innovations will be developed, deployed and scaled to tackle pressing challenges in agriculture and food systems in Africa. New and high-quality skills and capacity, learning opportunities and collaborations, and new policies and strategies will be developed through the interactions of science-policy-practice systems in the network to sustain a continued application of artificial intelligence research and innovations in transforming agriculture and food systems in Africa.

### 1.2 Importance of responsible and gender-inclusive AI innovation in agriculture

Agriculture in Sub-Saharan Africa is a vital industry involving a large portion of the population and playing a key role in ensuring food security and driving economic growth. Nevertheless, this region encounters diverse obstacles such as the impacts of climate change, insufficient availability of resources, and disparities in gender representation within the agricultural domain. The employment of artificial intelligence technologies offers a hopeful opportunity for addressing these obstacles by effectively utilizing resources, aiding data-driven decision-making procedures, and improving agricultural productivity. By using AI technologies, Sub-Saharan Africa can overcome these obstacles and enhance the promotion of sustainable agricultural practices to advance the continent. AI is revolutionizing global agriculture by enhancing various practices. Precision farming, integrating AI with farming data, boosts sustainability and profitability (Rosana and Rogério, 2023). The use of AI in agriculture covers soil and crop monitoring, predictive analytics, and agricultural robotics, improving productivity and sustainability (Mark et al., 2023). AI technologies like machine learning, IoT, robotics, and computer vision are pivotal in agriculture, with over 20 techniques applied globally (Kumar et al., 2023). Despite its potential, AI adoption in agriculture faces challenges due to diverse impacts, ethical concerns, technological disparities, and economic implications (Aiswarya et al., 2022). The evolving landscape of AI in agriculture emphasizes interdisciplinary collaboration for robust, economically valuable, and socially desirable solutions.

The use of AI in agriculture must be developed responsibly, to ensure that the solutions do not expand existing inequalities. It should be developed ethically and aligned with local contexts and community needs (Kumar, 2024). Responsible and gender-inclusive AI innovation in agriculture is crucial for addressing social, ethical, and gender-related concerns while enhancing productivity and sustainability (Maaz et al., 2022; Diana et al., 2022; Leo et al., 2021). AI technologies offer significant benefits in providing farm-specific recommendations, improving decision-making processes, and contributing to food security (Mark et al., 2023; Bart et al., 2019). However, the slow adoption of AI in agriculture is attributed to diverse impacts, ethical considerations, technological disparities, and economic implications. Integrating gender norms and local conditions into AI development can lead to more inclusive and sustainable agricultural practices. By fostering responsible innovation through inclusive processes and interdisciplinary collaboration, AI in agriculture can mitigate frictions, ensure equitable distribution of benefits, and enhance the overall well-being of farmers.

### 1.3 The landscape of AI applications in African agriculture

The landscape of AI applications in African agriculture is rapidly evolving, with a focus on enhancing productivity, reducing losses, and addressing food insecurity (Catherine and Hannah, 2023). AI technologies are being utilized for crop classification, yield estimation, pest monitoring, farm advisories, and disease detection, leading to decreased farmer losses and improved decision-making (Foster et al., 2023). Various AI techniques such as Neural Networks, Genetic Algorithms, and Support Vector Machines are being employed for disease forecasting, climate prediction, and weed detection, offering high accuracy and cost-effectiveness (Reena et al., 2022). However, there are concerns about Responsible AI implementation in African contexts, emphasizing the need to address issues of fairness, bias, and privacy to ensure the technology benefits all stakeholders. Additionally, there is a growing recognition of the potential for AI to reinforce hierarchies and disregard Indigenous knowledge in agriculture, highlighting the importance of inclusive governance and decolonial deliberation in shaping AI applications for African agriculture.

### 1.4 Objectives of the paper

This paper aims to present the experiences and lessons learned from the AI4AFS Innovation Research Network, highlighting the challenges faced by grantees in developing and deploying responsible AI solutions for agriculture and food systems. It showcases a diverse range of AI applications, including crop monitoring, pest detection, soil health assessment, and market forecasting, while exploring strategies for ensuring gender inclusivity, ethical considerations, and social inclusion. Furthermore, the paper provides actionable insights and recommendations for scaling up responsible AI innovation in agriculture, fostering an inclusive and just technology ecosystem, and contributing to sustainable development goals.

Specifically, the paper seeks to;

1. Examine Strategies for Inclusive Participation and Representation: This objective highlights the approaches employed by AI4AFS grantees to ensure inclusive participation and representation of marginalized groups, including women, smallholder farmers, and local communities. It directly reflects the program’s goal of prioritizing equity, fairness, ethical principles, and gender equality in AI solutions. The objective will also explore methods for engaging local stakeholders, conducting participatory design processes, and overcoming cultural barriers to technology adoption.
2. Explore Innovative Approaches to Overcoming Technical and Ethical Challenges: This objective aligns with the intervention of setting up and managing an artificial intelligence innovation research network. It discusses the innovative strategies employed by grantees to address data availability and quality issues, overcome infrastructure constraints, and integrate ethical principles into AI governance frameworks. Furthermore, it explores the potential for combining AI with emerging technologies such as the Internet of Things (IoT) and remote sensing to drive sustainable agricultural practices.
3. Provide Actionable Insights for Scaling and Sustaining Responsible AI Innovations: Reflecting the Hub’s aim to foster collaborations, knowledge exchange, and valorization within and beyond the network, this objective focuses on providing actionable insights and recommendations for scaling up responsible AI innovation in agriculture. It emphasizes the importance of developing context-specific and culturally appropriate AI solutions, ensuring their relevance and adoption within local agricultural contexts, and contributing to sustainable development goals at the community, regional, and national levels.

### 1.5 Research hypothesis

This study hypothesizes that responsible, gender-inclusive AI applications can boost agricultural productivity and climate resilience in Sub-Saharan Africa, providing a scalable framework for diverse global regions.

## 2 Methodology

### 2.1 Research design

This study employs a qualitative research design to explore the experiences and lessons learned by the 10 grantees within the AI4AFS Innovation Research Network. Given the complexity and context-specific nature of developing responsible AI solutions for agriculture and food systems, a qualitative approach is well-suited to capture the nuanced insights and diverse experiences of the participants. The research is designed as a multiple-case study, focusing on three grantees out of ten (10) across eight countries in Sub-Saharan Africa. This design allows for an in-depth exploration of the strategies employed by each grantee, the challenges they faced, and the outcomes of their projects. The sample size of three (3) was carefully determined based on the principles of qualitative research, where the emphasis is on depth rather than breadth. This study employed a purposive sampling approach to select participants who were directly involved in the AI4AFS Innovation Research Network, ensuring that the selected cases were rich in information and highly relevant to the study’s objectives. Prior to selection of participants, the network employed call for concept notes and call for proposals to select 10 consortia made up of research organizations, civil society organizations, and private sector organizations working at the intersection of AI, agriculture, food systems. The selection of these consortia were based on qualification and team composition, experience in relevant area of research and innovation, technical capabilities, impact pathway to achieving responsible AI for agriculture and food systems, governance structure, clear allocation of funds (budgeting). The selected grantees where trained through a series of co-created training and capacity building in need areas identified by the grantees. The grantees were trained in developing impactful research designs and methods, intellectual property rights in responsible ai, tracking carbon footprint in AI models, product development and commercialization of AI technology in agriculture and food systems, and development of policy brief and advocacy. These series of targeted training and capacity building on responsible AI and gender equality and social inclusion principles ensured that the network grantees project implementation was inclusive and responsive. Furthermore each consortia were guided to collect local datasets and engage the rural communities in there project problem definition and design to ensure that the solutions are community driven.

### 2.2 Justification for sample Size

- Data Saturation: Data saturation was achieved by conducting in-depth interviews and case studies until no new information or themes emerged. This point of saturation indicated that the sample size of three were sufficient to capture the full range of insights and experiences related to the development and deployment of AI solutions in agriculture and food systems. The iterative process of data collection and analysis ensured that all significant themes were thoroughly explored.
- Thematic Richness: Despite the small sample size, the diversity of the participants—spanning different countries, AI applications, and stakeholder roles—provided a broad spectrum of perspectives. This diversity enriched the thematic analysis, allowing for a comprehensive understanding of the challenges, strategies, and outcomes associated with responsible AI innovation in agriculture. The rich, detailed data obtained from these three cases provided a robust foundation for identifying key themes and patterns relevant to the study’s aims.

### 2.3 Data collection methods

Data were collected using a combination of semi-structured interviews, document analysis, and participant observation. The primary data source was semi-structured interviews conducted with the principal investigators of the grantees’ projects. Each interview was conducted either in person or via video conferencing, depending on the participants’ location and availability. The interviews focused on eliciting detailed accounts of the grantees’ experiences, including the challenges they encountered, the strategies they employed to overcome these challenges, and the outcomes of their AI interventions. Document analysis was also employed to review project reports, proposals, and other relevant documentation provided by the grantees. This method provided additional context and allowed for triangulation of the data collected through interviews. Participant observation was conducted during site visits to selected project locations, enabling the researchers to observe the implementation of AI solutions in real-time and to engage with the local communities involved. The combination of these data collection methods ensured a comprehensive understanding of the grantees’ experiences, while also allowing for the validation and cross-checking of information across different sources. All interviews were recorded with the participants’ consent and transcribed verbatim for analysis.

### 2.4 Data analysis

**Figure 1:**
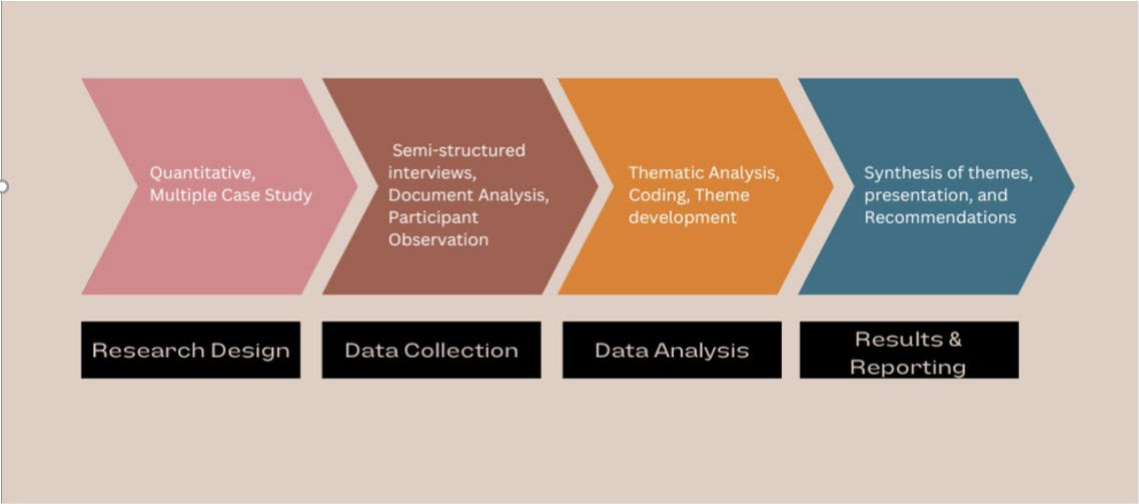
Flowchart for the study’s methodology.

The data analysis process was guided by thematic analysis, a method that allows for the identification and interpretation of patterns or themes within qualitative data. The analysis followed a systematic process beginning with data familiarization, where transcripts and documents were read multiple times to gain a deep understanding of the content. This was followed by the coding process, where significant portions of text were highlighted and assigned labels or codes that represent specific ideas or concepts. Codes were then grouped into broader themes that captured the key aspects of the grantees’ experiences and the challenges they faced. The themes were refined and organized into a thematic framework that reflects the study’s objectives, specifically focusing on strategies for inclusive participation, innovative approaches to technical and ethical challenges, and insights for scaling responsible AI innovations. Throughout the analysis, particular attention was given to the emergence of new themes that were not anticipated at the outset of the study, as these often provide valuable insights into the complexities of deploying AI solutions in diverse agricultural contexts. Data saturation was achieved as no new themes emerged after the analysis of the sixth case, confirming that the sample size was adequate (see Figure 1).

Methodological Limitations: This study acknowledges limitations in terms of scalability and applicability beyond Africa, noting the need for further research to address these challenges.

## 3 Results

### 3.1 Case studies: responsible AI solutions for agriculture and food systems

The AI4AFS Innovation Research Network gave sub-grants to 10 multidisciplinary teams made up of researchers, Private sector actors, and Civil Society Organizations. While the list of the 10 projects are presented below, the paper will present three case studies of responsible AI solutions.

The 10 research and innovation themes and countries of implementation include:

- Project 1: Monitoring and Artificial Intelligence Tools for Smart Agriculture—Cape Verde
- Project 2: Development of Machine Learning Model for Crop Pests and Diseases Diagnosis Based on Crop Imagery Data—Tanzania
- Project 3: Enhancing Farm-scale Crop Yield Prediction using Machine Learning for Internet of Agro – Things in Tanzania—Tanzania
- Project 4: Using Artificial Intelligence to enhance the Production, Management and Marketing of Nsukka Yellow Pepper (*Capsicum Chinense* Nsukkadrilus)—Nigeria
- Project 5: Scaling Smartphone-Based Tools for Early Crop Diseases Detection and Monitoring—Uganda
- Project 6: Pest Occurrence Early Warning System and Diagnostic Tool Development using Geoinformation and Artificial Intelligence: A Case Study of Tomato Leaf Miner (*Tuta absoluta*), and Whiteflies in Machakos County, Kenya)—Kenya
- Project 7: Empowering Smallholder Farmers (SHF) in Busia County using Low-Cost IoT (Internet of Things) and AI (Artificial Intelligence) Tools—Kenya
- Project 8: Building the artificial intelligence (AI) for soil moisture and nutrient monitoring under irrigated agriculture among smallholder farmers, academic and agriculture experts in Malawi—Malawi
- Project 9: TOLBI AI, an AI-based digital tool for smart, sustainable, and efficient agriculture—Senegal
- Project 10: Detection of Crop Pests and Diseases on Web and Mobile Devices using Deep Learning—Ghana

### 3.2 Case study 1: pest occurrence early warning system and diagnostic tool development using geo-information and artificial intelligence: a case study of tomato leaf miner (*Tuta absoluta*), and whiteflies in Machakos County, Kenya

#### 3.2.1 Project overview and objectives

The Kenya Horticulture sub-sector is the largest in agriculture contributing 33% of the agricultural GDP (Salome et al., 2020). The subsector has been successful in the past three decades, offering the best alternative for among others, increased food self-sufficiency, food security, and improved nutrition, all ensuring the generation of increased incomes and employment, and foreign exchange earnings. The threats of climate change have however affected both productivity and profitability of the sector, resulting to limited growth and sustainable development. Increasing temperatures and changes in atmospheric moisture have resulted in the emergence of new pests as well as an upsurge of existing ones. Tomato is one of the crops affected by changes in weather, and according to the Government of Kenya it accounts for 14% of total vegetable production and 6.72% of total horticultural crops (Sigei et al., 2014). In Kenya, the tomato plays an important role in meeting domestic and nutritional food requirements, job creation, income generation and foreign exchange earnings, thus contributing to poverty alleviation. Despite its importance, tomato production is constrained by pests and diseases accounting to 80–100% losses, with the most common pests being tomato leafminer (*Tuta absoluta*) and white flies. Tomato leafminer can cause 100% damage in tomato crop in both greenhouses and open fields if control measures are not carried out. The pests reduce crop quality, increasing tomato prices and regional bans on the trade of tomato including seedlings. Therefore, the research was focused on developing an AI-based spatial tool for the monitoring and surveillance of *Tuta absoluta* and whiteflies on tomato crop in Machakos County, Kenya. The team utilized remote sensing deployed to monitor changes in land surface temperatures and atmospheric moisture to enable real-time monitoring of pest occurrence. Artificial intelligence was used to mine data that can be used for the identification and control of these pests. The spatial tool developed was sustainably implemented among smallholder farmers through the Kathaana vegetable growers in a business model that involves the youth and women for e-extension services. The framework was designed to focus on early identification of pests and control through an integrated solution system inbuilt into the tool for varied users with limited pest control knowledge.

#### 3.2.2 AI solution developed

**Figure 2:**
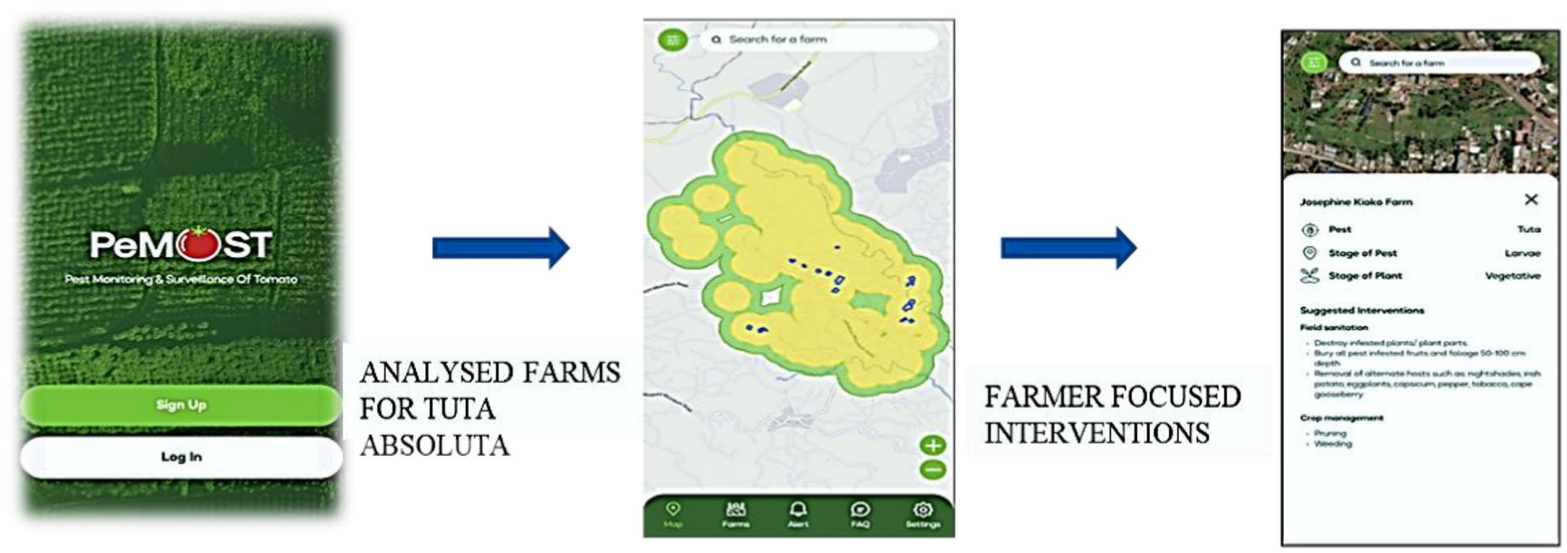
The PeMOST application for early monitoring and surveillance of *Tuta absoluta (Leaf Miner).*

The Pest Monitoring and Surveillance of Tomato Tool (PeMOST) was developed by the team of researchers from Geo Spatial Research, Jumo Kenyatta University of Agriculture and Technology, and Kathaana Vegetable Growers, Kenya. Figure 2 shows a sample illustration of the PeMOST Tool. The tool is a spatial-based AI system for early warning. It uses satellite images and compares them with historical data using AI algorithms and detect that if there is significant changes in temperature for presence of pest; and send alerts to farmers to their smartphones so that farmers can take required precautions and use required pest control thus AI helps farmers to fight against pests.

### 3.3 Strategies for ensuring gender inclusivity and ethical considerations

**Figure 3:**
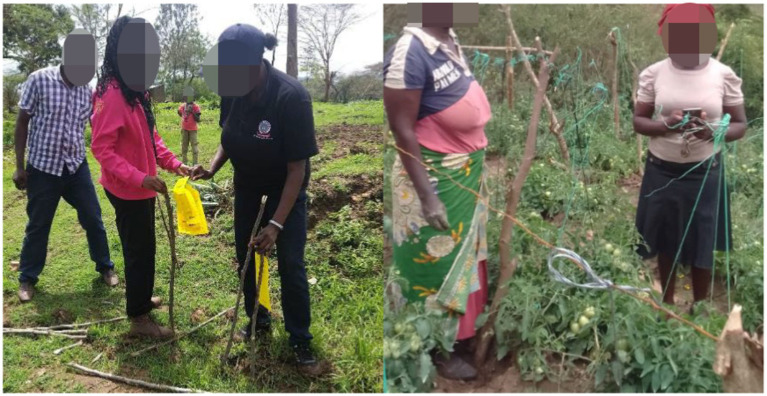
An inclusive data collection on *Tuta absoluta* by women and males.

There were deliberate efforts to engage all genders in this project from the conceptual stage all through to the final stage. No adjustments were made in the course of the implementation. Gender inclusivity was demonstrated by the composition of the implementing team, enumerators, and project stakeholders. The core implementing team was composed of three ladies and three gentlemen with the Principal Investigator (PI) being a lady. The developers’ team was also composed of two gentlemen who were in charge of the frontend and the middleware respectively, and one lady who was in charge of the back-end development. To ensure that the project is need driven and of top priority to the women and marginalized, a baseline survey was conducted, where 95% of the respondent acknowledged *Tuta absoluta* as great problem in the farm and 97% of the farmers used chemical pesticides for control of *Tuta absoluta*. During the field surveys, there were deliberate efforts to involve both male and female enumerators in data collection, where 40% were ladies and the remaining 60% were male. Given that these enumerators were also local, it was expected that they would provide a clear gender lens and bring to the fore any gender issues that might affect the project implementation and not captured by the questionnaires. To ensure further gender inclusivity and streamline this among the project beneficiaries, who are about 200, there is a sampled gender distribution of 36% female and 64% male distribute across the different age brackets that was surveyed. The different gender plays a critical role in the day-to-day management of their farms and consequently adoption of technologies proposed by the project. During project meetings, the invitations targeted both female and male beneficiaries to enhance gender inclusivity. This not only covered attendance but also participation. During discussions, the female and male views were integrated into the proceedings as captured in the meeting minutes. Figure 3 shows gender integration in data collection which ensured responsible AI development.

#### 3.3.1 Challenges faced and lessons learned

The main challenge faced during the implementation of the project was access to quality satellite data during the PeMOST tool development. Data plays an important role in ensuring there are no bias in the AI development. Another serious challenge was access to internet service especially in the rural communities in Kenya. Data services need to be improved to ensure that the tolls are effectively used by the famers. Smart phones play a major role in the use of AI mobile applications; most rural women lack access to smart phones and lack the literacy of using such smart phones. To address these challenges, future projects should prioritize securing high-quality satellite data, acquiring ground truth data from various sites, ensuring reliable internet connectivity, and providing smartphone literacy training, especially for rural farmers.

Teamwork and collaboration across different institutions are vital for success, along with early planning and proper organization are opportunities that can be tapped. Moreover, stakeholder engagement is crucial for building synergies and ensuring successful project implementation. Farmers are eager to adopt AI tools when there is a strong collaboration with them from development to the deployment of such tools. Such partnership ensures that initial fears inherent in the introduction of new technology are removed and confidence built with the end-users. Another key lesson is the adoption of ICT tools are increased when such technologies are introduced with other solutions that assist the farmer in farm production. The adoption of the PeMOST tools increased due to the introduction of water trap and pheromones a biological control method for *Tuta absolut*a.

### 3.4 Case study 2: detection of crop pests and diseases on web and mobile devices using deep learning, Ghana

#### 3.4.1 Project overview and objectives

The University of Energy and Natural Resources, Ghana Developing Communities Association, and DIGILECT System a private sector technology actor worked together to solve natural resource problems through interdisciplinary research in Science, Engineering, and Agriculture. The communities in the University’s catchment area are highly dependent on agriculture for their livelihood. They feed their families with food from maize, cassava, and tomatoes and sell some for financial gains. Due to the scarcity of land, the farmers cultivate cashew plants on small pieces of land to support their families financially. However, these crops are infested by pests and diseases every farming season, resulting in crop losses, hunger, malnutrition, low income, and poverty. To contribute to the improvement of farmers’ livelihood, we aim at developing a deep learning (DL)-based mobile (Android/iOS) and web app to efficiently detect cassava, maize, tomatoes, and cashew pest/diseases. The DL models will be trained on Google Collab (to reduce carbon footprint) with both healthy and sick images of the plants. The models will be embedded in a mobile app and deployed on mobile phones using the Tensor Flow lite framework. When the app is installed on a mobile device, the user may capture/scan a plant with the phone’s camera and the recognition will be shown instantaneously in addition to the certainty (probability value) of the results. For further verification of high uncertain outputs (identified pest/disease with high uncertainty), the user will be alerted of the necessity to seek clarification from an expert serving as a man-in-the-loop for the system. Due to high illiteracy rates in the farming communities, our AI system will be user-friendly and have text-to-voice facility to communicate the results and recommendations in English, the popular local language “Twi” and “Akan.” This is to also facilitate easy usage by the visually impaired. To achieve responsible AI, the models will be designed to be privacy preserving and robust by means of frequent security updates. Due to low internet penetration, the mobile apps will not need internet connectivity for detecting plant pests/diseases. As part of gender equality and inclusion, the e-kiosks will be managed by women and disabled persons. To increase adoption of the tool, Farmers were trained on local methods of controlling plant pests and diseases using natural ingredients such as the solution obtained from leaves of a “nim” tree being very effective for controlling fall army-worms in maize. The web app is available at https://www.uenrai4afs.app/.

#### 3.4.2 AI solution developed

**Figure 4:**
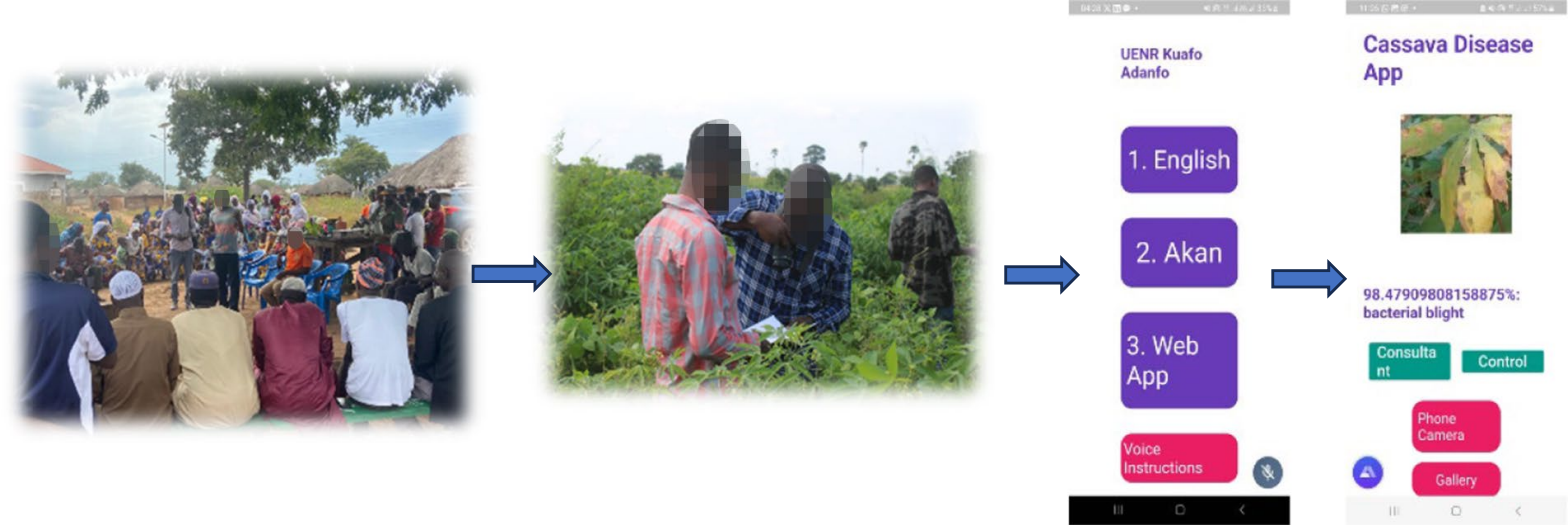
The mobile and web AI based multi-crop disease detection tool.

The project team from Ghana developed an AI enabled mobile and web apps to help farmers to detect crop diseases/pests, specific crops handled by the AI tool are cassava, maize, tomato, and cashew. This has the potential to help reduce crop losses, reduce malnutrition, and improve income of farmers. Figure 4 shows a view of the mobile/web app developed by the team in Ghana. The app has been shared with over two thousand (2,000) farmers through their WhatsApp group and one thousand (1,000) farmers have also installed the app through the assistance of the e-kiosk attendants. User feedback indicated that the app achieved 85% accuracy in detecting plant diseases, with farmers reporting a 15% reduction in crop loss due to improved early detection of disease.

#### 3.4.3 Strategies for ensuring social inclusion and community engagement

**Figure 5:**
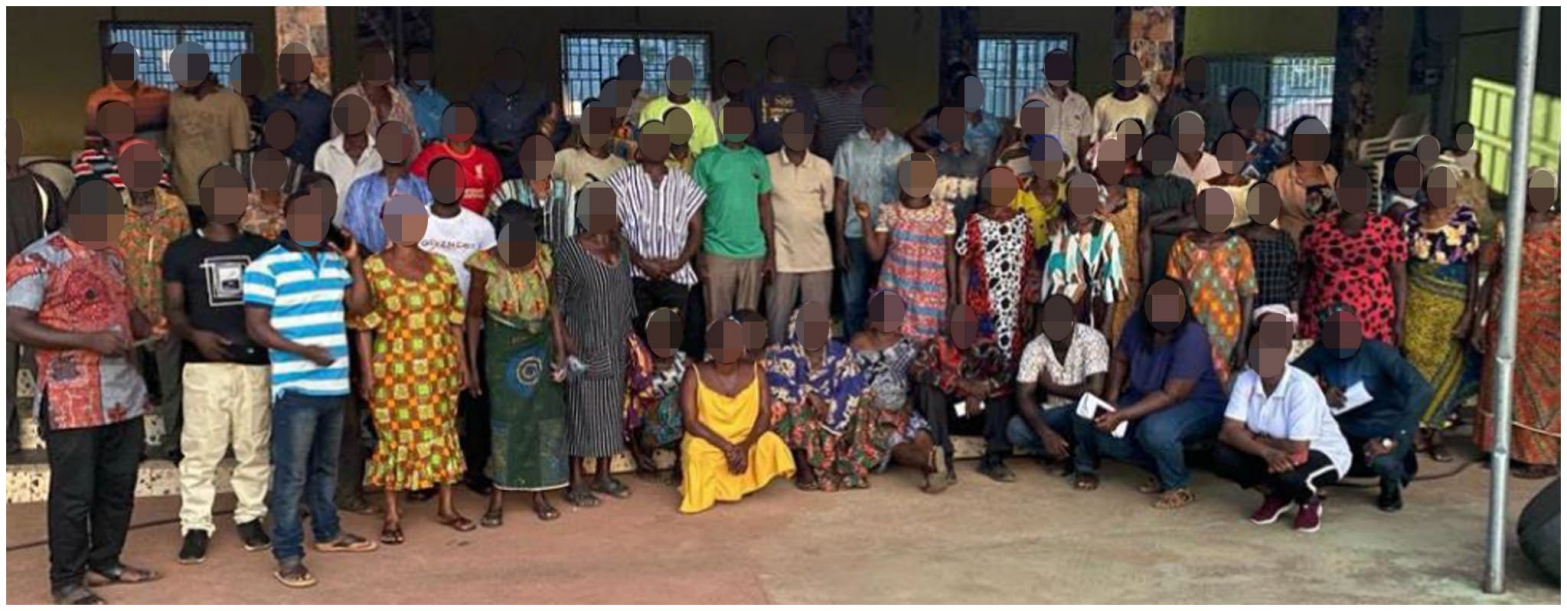
User training at Baamire (Bono East Region of Ghana) on the use of the AI disease detection App.

To ensure that gender and diversity issues are addressed to satisfactory levels, a gender and diversity sub-committee was established to monitor project activities such that they do not infringe on the rights of/or marginalize any group of people. The sub-committee ensured that beginning with role assignment at the team level, they provided equal access, resources, opportunities, benefits, and rights for males and females, people with disability, as well as people with different religious orientations. As a strategy, we prioritized the involvement of females and the disabled during the initial stakeholder engagements to enable us to achieve equitable outcomes for different groups. To encourage change in the perceptions that the major role of females is in the kitchen, we prioritized and reached out to female farmers to collect data on their farms to create a form of partnership and give them the feeling of belongingness and importance in their chosen profession. Moreover, to ensure high level of adoption, the farmers were engaged from the time of project conception to the deployment of the developed tools. Series of community training and engagement took place with the farmers drawing from the experiences of the Ghana Developing Communities Association, a non-governmental organization partnering with the team. Figure 5 shows a group photograph of the women, men, youths, and PLWDs trained in the use of the mobile/web application.

#### 3.4.4 Challenges faced and lessons learned

The project implementation saw that stakeholders, especially farmers show resistance to providing information to researchers who intend to do research due to past experiences with researchers. They claim to have received many of these researchers over the past, yet their lives have not changed. Afro-centric large datasets are needed to train deep learning models to avoid bias and overfitting. The cost of data acquisition is very high necessitating the development of open source data repositories. Most of the datasets that are used to train deep learning models are generated outside Africa. Training deep learning models on the Internet (e.g., Google Collab) requires a very fast Internet that is available and also reliable. The Internet availability in most rural communities is poor, hence the use of internet-based mobile apps is limited, thus a challenge to large-scale deployment of Ai mobile apps. The cost of acquiring a license for cloud-based services like google colab is very high. Failing to address the internet issue could result in multiplying the carbon footprint; that is, emissions from the local machine plus that from the remote GPU device. The development of AI algorithms contributes to green gas emissions to the environment. A serious challenge is to track the carbon footprint of such training requiring a lot of capacity building among tech developers.

### 3.5 Case study 3: using artificial intelligence to enhance the production, marketing, and Management of Nsukka Yellow Pepper in Nigeria

#### 3.5.1 Project overview and objectives

The Association of Professional Women Engineers of Nigeria (APWEN), Educare Tech company, and the University of Nigeria, Nsukka synergized to use artificial intelligence in providing solutions to agricultural problems. The project “Using Artificial Intelligence to Enhance the Production, Marketing, and Management of Nsukka Yellow Pepper in Nigeria” addresses critical challenges faced by rural farmers, particularly women, in the production, management, and marketing of this economically valuable crop. Nsukka Yellow Pepper (NYP) stands out in the market due to its unique aroma, yellow color, and high capsaicin content, attracting significant demand. However, its cultivation is plagued by various constraints, including soil nutrient depletion, climate change-induced water scarcity, disease and pest infestation, high input costs, inadequate extension services, and limited market access. The overall objective of the project is to leverage artificial intelligence (AI) tools and applications to improve the productivity and yield of NYP by strengthening the resilience of farmers in adapting to climate change, applying mitigation strategies, and thus empowering the women and youths. Specifically, the project addressed these challenges by collecting datasets for early disease detection, supporting soil nutrient monitoring, improving water conservation through smart irrigation, and providing e-extension services to farmers. The participatory action research approach was adopted, involving stakeholders in co-developing and deploying responsible AI solutions tailored to the needs of rural farmers.

Key methods and approaches used in the project include the collection of datasets of NYP, annotation, and classification of the datasets, these annotated datasets were partitioned into training, validation, and test sets using the provided train_val_test_split.py script. Deep learning algorithms were developed using Python 3.8. TensorFlow served as the core deep learning framework, while NumPy facilitated numerical operations. OpenCV played a crucial role in image processing, and Matplotlib aided in visualizing results. CodeCarbon, an emissions tracking library, was incorporated to assess the environmental impact of model training. The project also prioritizes gender equality and inclusion, ensuring that women, youth, and marginalized groups are empowered to adopt and utilize AI technologies in farming practices. Marginalized groups refer to populations that face systemic exclusion or disadvantage due to historical inequalities, discrimination, and structural barriers. In the context of technology and AI, these groups are often underserved due to biased datasets, lack of representation in design processes, and the digital divide, which limits their access to and benefits from technological advancements. These challenges necessitate deliberate efforts to ensure inclusivity and equity in AI adoption.

The project has successfully concluded with the development and implementation of three major components aimed at addressing critical challenges faced by rural farmers, particularly women, in NYP production. Firstly, the project has developed AI-based disease detection systems capable of identifying and classifying two class-level (healthy and unhealthy) Nsukka yellow pepper crops. These systems utilize deep machine learning algorithms to analyze images of pepper plants, enabling early detection and targeted intervention to mitigate disease outbreaks. Secondly, an IoT-based nutrient monitoring and smart irrigation system has been deployed to enhance soil fertility management and optimize water use in pepper cultivation. This system leverages sensor technology to monitor soil nutrient levels and moisture content in real-time, providing farmers with valuable insights to adjust irrigation schedules and fertilizer application, thus improving crop health and productivity. Furthermore, the project has developed and launched the “APWEN Farm App,” an e-extension application available on the Google Play Store. This app serves as a comprehensive digital platform for delivering extension services, providing farmers with access to agronomic advice, market information, weather forecasts, and training resources, empowering them to make informed decisions and improve their farming practices. In addition to these technological innovations, the project has facilitated a policy dialogue involving all stakeholders, including government agencies, academia, industry partners, civil society organizations, and farmers’ cooperatives. This dialogue served as a platform for discussing key issues and policy recommendations related to AI adoption in agriculture, fostering collaboration and knowledge exchange among diverse stakeholders. Moreover, the project has established a greenhouse facility to demonstrate best practices in NYP cultivation and serve as a training center for farmers. Additionally, the project has facilitated the registration of women farmers in a cooperative, promoting collective action and empowerment within the farming community. Overall, the project has made significant strides in enhancing the resilience, productivity, and market access of rural farmers engaged in NYP production. By harnessing the power of artificial intelligence, IoT technology, and digital extension services, the project has contributed to sustainable agricultural development, gender empowerment, and economic. The deployment of these solutions led to 50% marginal increase in the area of field cultivated as farmers as the use of the technology boasted farmers’ confidence to produce more crops resulting in higher crop yields and about 30% reduction in pest infestations because of early detection. A cooperative society called “Nwanyibuihe” that “Woman is Valuable” cooperative society was established as a result of the co-creation approach.

#### 3.5.2 AI solution developed

**Figure 6:**
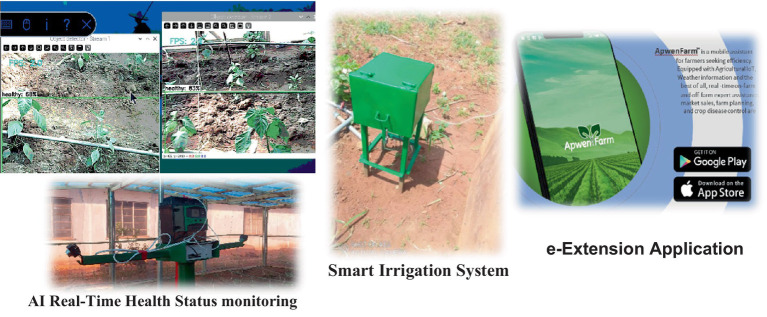
The technological solutions developed in Nigeria for small-scale farmers.

Working with the farmer communities, the project team developed and deployed three unique solutions that were needs-driven. The solutions include an AI real-time on-farm disease detection system deployed on Raspberry Pi4, Text message-activated IoT-Based Irrigation system for efficient water management, and e-extension application that links the farmers to the extension agents. Figure 6 shows the developed tools.

#### 3.5.3 Strategies for ensuring gender equality, social inclusion and community engagement

**Figure 7:**
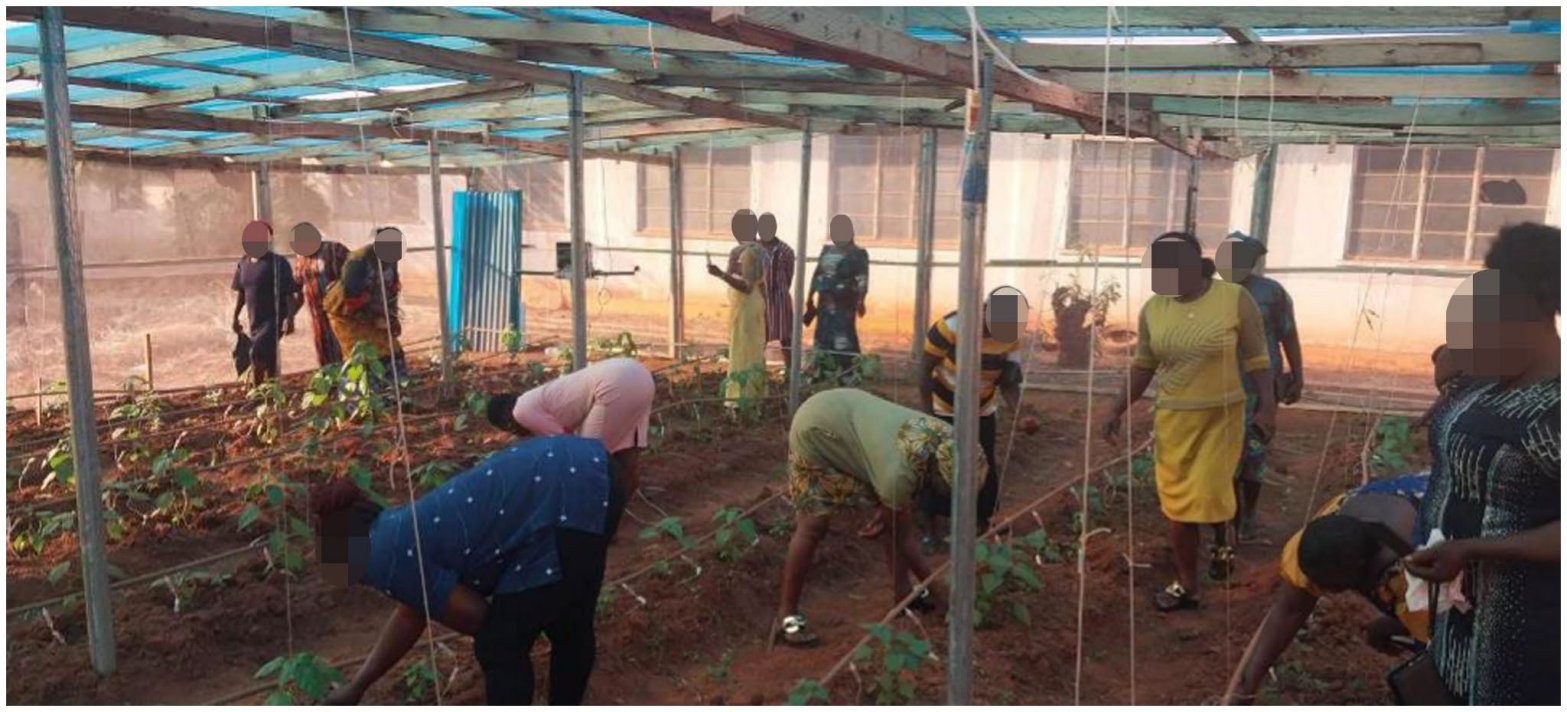
The onsite retraining of the farmers on installing the AI disease detection system in a demonstration farm.

**Figure 8:**
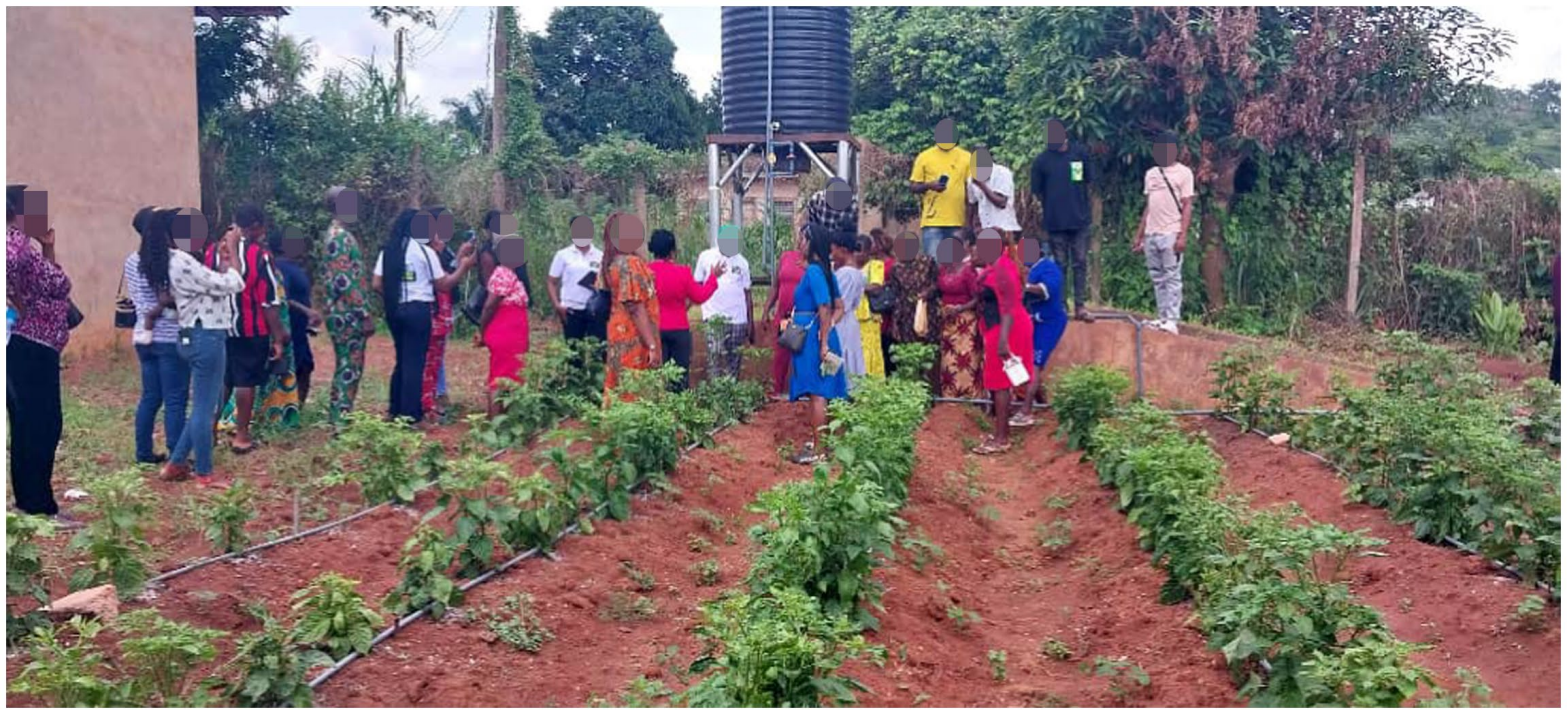
The onsite training of the farmers on installing the IoT-based smart irrigation system in a demonstration farm.

The project followed the GEI principles ensuring an equal learning and discussion environment in all its activities. From the start of the project, the team engaged with the women, men, youth, and people with disabilities in understanding their needs and prioritizing these needs in the design of the solutions. To ensure gender equality and inclusion, the composition of the team is made up of 4 women and 4 men. The PI and co-PI are women, while the private sector co-PI is a Man. equal opportunities in the activities. The Consortium upheld the principles of open dialogue and consultations within the research network. The consortium sought and received consent from community leaders before entering the community for community awareness and data collection. Female research assistants were given priority during recruitment to build their capacity in AI development and deployment. Gender issues were mainstreamed in all the project activities: Men, women, youth, girls, persons with disabilities (PLWDs), and marginalized groups had equal opportunities in the activities. The seating arrangement for discussion was such that all participants were relaxed and free to interact. The collection of gender-disaggregated data was prioritized in the research network to increase the likelihood of achieving gender-sensitive outcomes. The e-extension service app used symbols and logos that are gender inclusive. Crop datasets were collected by male and female research assistants in conjunction with the farmers. During co-development, deployment of the tools and policy dialogue, the team made sure that women and men, including the youths and PLWDs were involved. This is seen in Figures 7, 8, showing the onsite training on the use of the smart irrigation system and the disease detection system. During the onsite training both women and men were allowed to express themselves, ask questions, and give suggestions on ways to adapt the technology to their farms. Some suggested that they would require financial incentives to establish such a facility in their farm, while others accepted the idea of small clusters of 3–4 farmers using a facility through mutual contribution. Farmers reported a 40% improvement in water use efficiency, increase in hectare of cultivation from 1 or 2 hectares to 4–5 hectares, and expressed high satisfaction with the training, with 30% of participants adopting the new irrigation methods.

#### 3.5.4 Challenges faced and lessons learned

The project team faced some challenges that included the low reach of internet infrastructure in rural communities, the low level of access to android smart-phones. Subsequently, there were lessons learned in the project. The project underscores the importance of engaging local communities, stakeholders, and end-users throughout the design, development, and deployment of AI innovations. By involving them in decision-making processes and co-creating solutions that address their specific needs and challenges, the project has fostered greater acceptance, ownership, and sustainability of the innovations. For AI to be responsible there must be collection of quality datasets for training the AI models in order to avoid bias. The project has highlighted the importance of capacity-building and knowledge-sharing initiatives in driving the adoption and scalability of AI innovations. By providing training, technical assistance, and educational resources to farmers, extension agents, and other stakeholders, AI tools will be of immense benefit in increasing food production.

## 4 Discussion

### 4.1 Key challenges and lessons learned

**Figure 9:**
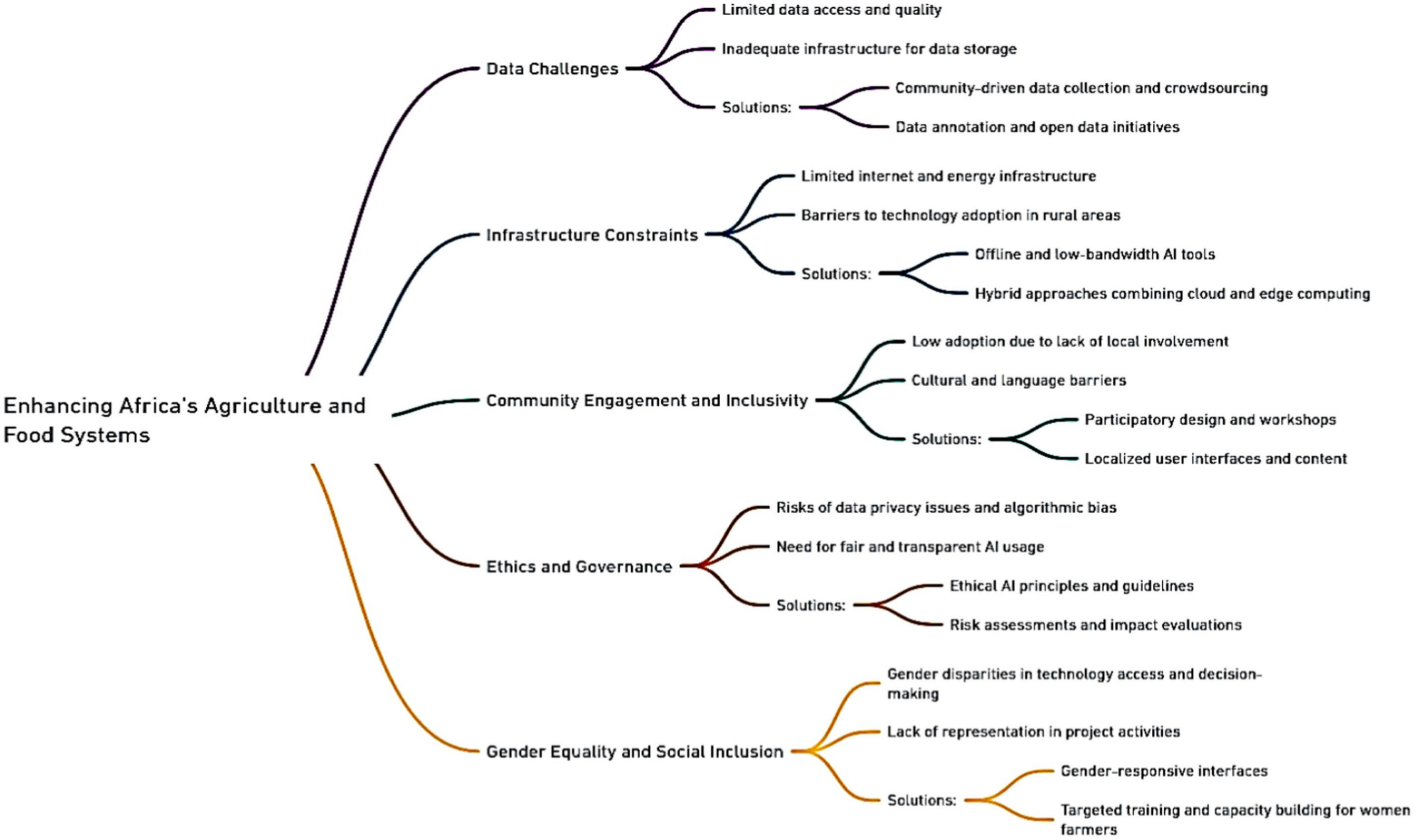
Challenges and solutions for enhancing Africa’s agriculture and food systems.

The implementation of the Artificial Intelligence for Agriculture and Food Systems Innovation Research network activities brought the project team to see the key challenges facing the use development, deployment, and scale of AI tools in Africa. It also provides insights into opportunities that emerge in adopting responsible AI in Africa. This section discusses some of the key challenges and opportunities for improving the rapid development and deployment of AI in Africa’s agriculture and food systems. Figure 9 shows the summarized challenges and solutions for enhancing Africa’s Agriculture and Food Systems (AFS) using artificial intelligence tools.

#### 4.1.1 Data availability and quality issues

Data issues affect the increased development of AI tools in Africa. These challenges include; limited access to structured data, poor data quality, inadequate data storage, lack of locally contextualized data, and lack of data regulatory policies; this is collaborated by studies by James (2022) and Gregory (2023). AI algorithms heavily rely on data for training and decision-making, and the absence of adequate data can limit the effectiveness and accuracy of the solutions. Additionally, there are difficulties related to insufficient infrastructure and network connectivity, which are essential for AI technologies that require a good quality connection and considerable energy requirements (Gregory, 2023). Moreover, the lack of a skilled workforce in AI due to shortcomings in the education system further hinders the development and deployment of AI tools in Africa. These data-related challenges collectively impede the full realization of the potential benefits of AI technologies in the African context. To address data availability and quality issues for enhancing AI development in Africa, the AI4AFS network developed a data repository for open access to data collected by the grantees during the implementation of their projects. Therefore, there is a need for improved access to data and better utilization of existing data for program enhancements (Ade-Ibijola and Okonkwo, 2023). The following are key emerging opportunities:

- Data collection and crowdsourcing: There is an opportunity to leverage crowdsourcing and community-driven data collection efforts to build locally relevant and contextualized datasets. By engaging farmers, extension workers, and local communities in data-gathering processes, stakeholders can tap into indigenous knowledge, local practices, and on-the-ground observations. By leveraging crowdsourcing, farmers can contribute real-time data on agricultural practices, challenges, and outcomes, which can be utilized to develop AI solutions tailored to their specific needs (Kumar et al., 2023; Adem and Abebe, 2022). Additionally, crowdsourcing can help in gathering data on factors influencing the adoption of AI technologies, such as training availability, access to information, and proximity to AI centers, as highlighted in studies focusing on AI adoption in dairy farming in Ethiopia and Tanzania (Kabuni et al., 2023; Musungwini et al., 2023; Awuor and Rambim, 2022). This collaborative approach not only enhances the development and customization of AI tools for smallholder farmers but also fosters knowledge sharing and community engagement, ultimately promoting sustainable agricultural practices and improving livelihoods in the region.
- Data annotation and labeling: With the rise of machine learning and computer vision techniques, there is a growing need for high-quality, annotated datasets to train AI models effectively. This presents an opportunity for collaboration between AI researchers, domain experts, and local communities. By involving stakeholders in the data annotation and labeling process, stakeholders can ensure that the data is accurately labeled, accounting for local nuances and cultural contexts.
- Open data initiatives and data sharing: Promoting open data initiatives and data sharing platforms can facilitate access to diverse and representative datasets. By encouraging the sharing of agricultural data among researchers, organizations, and governments, stakeholders can leverage existing data resources and reduce redundant data collection efforts (Sunny et al., 2021). This can accelerate the development of AI solutions and foster cross-regional collaborations. ATPS and partners developed a knowledge-sharing platform for data storage in line with the open access policy.
- Data quality assurance and standardization: Developing robust data quality assurance frameworks and standardization protocols presents an opportunity to enhance the reliability and interoperability of agricultural data. By establishing guidelines for data collection, storage, and processing, stakeholders can improve data consistency, reduce errors, and ensure that AI models are trained on high-quality and reliable data. For instance, in Kenya, inconsistent satellite imagery quality affected pest monitoring, which was mitigated by integrating local farmer-reported data to enhance accuracy.
- Integration of traditional/indigenous knowledge: There is an opportunity to integrate traditional and indigenous knowledge systems with modern data collection techniques. By documenting and digitizing local agricultural practices, environmental observations, and traditional ecological knowledge, stakeholders can create valuable data sources that can complement and enhance AI models, ensuring their relevance and applicability to local contexts; and
- Increasing Public-private partnerships: Fostering public-private partnerships can facilitate data sharing and access to proprietary datasets held by private companies or research institutions (Abdul-Rahim, 2022). By establishing mutually beneficial agreements and collaborations, stakeholders can leverage diverse data sources, combine complementary datasets, and drive innovation in AI for agriculture.
- Leveraging existing open-source datasets and integrating them with local data: Leveraging existing open-source datasets and integrating them with local datasets can significantly increase the availability and use of Artificial Intelligence (AI) among smallholder farmers in Africa. Open-source datasets provide a wealth of labeled examples for training AI models (Cob-Parro et al., 2024). By integrating and customizing these datasets with locally collected data, such as crop yield records, weather patterns, and soil quality, AI models become more robust and accurate. Improved training data leads to better predictions and recommendations for farmers. Open-source datasets often lack region-specific information. Integrating local data ensures that AI models consider unique factors relevant to African smallholder farming, such as crop varieties, pests, and cultural practices. Customized models perform better in local contexts (Cob-Parro et al., 2024). Using open data sets customized with locally available datasets, the burden of collecting datasets from scratch is reduced, addressing data scarcity. It is also useful to use transfer learning by using pre-trained models from open-source and fine-tuning these models with local data allows them to adapt to specific agricultural conditions.

#### 4.1.2 Overcoming infrastructure constraints and technology adoption barriers

The successful deployment of AI solutions in agriculture often requires robust infrastructure, including reliable internet connectivity, access to computational resources, and the availability of appropriate hardware and software. However, many rural areas in Africa face infrastructure deficits, posing significant challenges to technology adoption. The AI4AFS team saw opportunities in the following areas to boast infrastructure gaps:

- Developing offline and low-bandwidth solutions: Developing offline and low-bandwidth solutions to address challenges faced by AI tool users in remote African communities is crucial (Rehman et al., 2018). The lack of broadband and energy infrastructure in these areas hinders the operation of AI technologies (Rehman et al., 2018). To overcome this, solutions like adapting AI systems to function offline and optimizing them for low-bandwidth environments are essential. Additionally, initiatives focusing on infrastructure development, such as providing reliable energy sources and improving connectivity, are vital (Rehman et al., 2018). These efforts align with the need to consider the socio-cultural implications of AI in Africa to ensure equitable access and utilization. To mitigate the limitations of poor internet connectivity, grantees focused on developing AI solutions that can operate in offline or low-bandwidth environments. These solutions leverage edge computing and local data processing capabilities, minimizing the need for constant internet access. By caching data and models on local devices or servers, farmers and agricultural stakeholders can access AI-powered services and decision support systems without relying on high-speed internet connections (Rehman et al., 2018). Additionally, these solutions can be designed to operate on low-cost hardware, making them more accessible and affordable for resource-constrained communities.
- Leveraging cloud computing and edge computing technologies: While cloud computing presents opportunities for scalable and cost-effective computational resources, its adoption may be hindered by limited internet connectivity in rural areas (Humphrey et al., 2018). To address this challenge, there is need to explore hybrid approaches that combine cloud computing with edge computing technologies. Edge devices, such as sensors and local gateways, can preprocess and filter data before transmitting it to the cloud, reducing bandwidth requirements (Omojokun and Segun, 2017). Additionally, edge computing enables real-time decision-making and response, which is crucial for time-sensitive agricultural applications like precision irrigation or pest monitoring (Tshepho et al., 2019).
- Providing training and capacity-building programs for end-users: Effective technology adoption requires not only infrastructure but also human capacity development. Grantees recognized the importance of providing comprehensive training and capacity-building programs for end-users, including farmers, extension workers, and agricultural professionals. These programs covered topics such as digital literacy, using AI-powered tools and applications, interpreting data and insights, and integrating AI solutions into existing agricultural practices. By building local capacity and fostering a culture of continuous learning, grantees aimed to empower communities and increase the likelihood of sustained technology adoption.
- Fostering public-private partnerships for infrastructure development: Overcoming infrastructure constraints often requires significant investments and long-term planning. Grantees explored public-private partnerships as a means to leverage resources and expertise from different stakeholders. By collaborating with governments, telecommunications companies, and technology providers, grantees could contribute to the development of rural infrastructure, such as internet connectivity, power grids, and computing facilities. These partnerships not only addressed immediate infrastructure needs but also laid the foundation for future technology adoption and scalability.

#### 4.1.3 Engaging local communities and ensuring inclusive participation

Effective community engagement and inclusive participation are crucial for the successful adoption and sustained impact of AI solutions in agriculture. Some opportunities for engaging local communities and ensuring inclusive participation include:

- Conducting participatory design workshops and focus group discussions: From the project implementation, the team recognized the importance of co-creating AI solutions with local communities to ensure their relevance and acceptance. Participatory design workshops and focus group discussions were conducted to understand the unique challenges, needs, and perspectives of different stakeholder groups, including smallholder farmers, women farmers, indigenous communities, and rural youth. These participatory processes enabled grantees to gather valuable insights, incorporate local knowledge, and tailor their AI solutions to the specific contexts and priorities of the communities they served.
- Engaging community leaders and local organizations: Building trust and fostering buy-in within local communities are essential for successful technology adoption. The team actively engaged with community leaders, elders, and local organizations, such as farmer cooperatives or women’s groups. By involving these influential stakeholders in the project planning and implementation stages, grantees could leverage their knowledge, networks, and credibility within the communities. This approach helped to address potential cultural barriers, build community ownership, and ensure that the AI solutions were aligned with local values and practices.
- Developing user-friendly interfaces and localized content: To promote inclusive participation, tools should be developed to focused on having user-friendly interfaces and localized content for their AI solutions. This involved designing intuitive and visually appealing interfaces that catered to varying literacy levels and technology familiarity. Additionally, translating content into local languages and incorporating familiar visual elements and metaphors helped to bridge the digital divide and increase accessibility for diverse user groups.
- Ensuring representation and participation of marginalized groups: The AI4AFS network recognized the importance of addressing potential biases and ensuring that marginalized groups, such as women, youth, and indigenous communities, were actively involved in the development and deployment of AI solutions. Targeted outreach efforts, capacity-building initiatives, and inclusive decision-making processes were implemented to amplify the voices and perspectives of these groups. By fostering diverse representation and participation, grantees aimed to create AI solutions that addressed the unique challenges and needs of marginalized groups, promoting equitable access and benefits.

#### 4.1.4 Integrating ethical principles into AI governance frameworks

As AI technologies become more prevalent in agriculture, it is essential to establish governance frameworks that ensure ethical and responsible development and deployment. The projected team believes that can be done by:

- Developing AI ethics guidelines and principles: To ensure the responsible use of AI in agriculture, the AI4AFS team developed comprehensive AI ethics guidelines and principles tailored to the local contexts and values of the communities they served. These guidelines covered key ethical considerations such as transparency, accountability, privacy, and fairness. They provided a framework for addressing potential biases, ensuring data privacy and security, and mitigating the risks of unintended consequences. By involving diverse stakeholders, including ethicists, policymakers, and community representatives, grantees aimed to create guidelines that reflected the unique cultural and social norms of different regions.
- Conducting ethical risk assessments and impact evaluations: The project team recognized the importance of proactively identifying and assessing potential ethical risks associated with their AI solutions. Conducting ethical risk assessments to analyze the potential impacts on various stakeholder groups, including marginalized communities, smallholder farmers, and the environment, is very important in developing responsible AI systems. These assessments help to uncover potential biases, privacy concerns, or unintended consequences that could arise from the deployment of AI systems. Additionally, there is need to implement impact evaluation frameworks to continuously monitor and assess the ethical implications of their AI solutions throughout the development and deployment phases.
- Establishing AI governance committees and advisory boards: To ensure ongoing oversight and accountability, the project teams found it necessary to establish AI governance committees and advisory boards. These multidisciplinary bodies will be made up of experts from various fields, including AI researchers, domain experts, ethicists, policymakers, and community representatives. AI governance committees will play a critical role in ensuring ethical AI development and deployment by setting clear standards for data privacy, algorithm transparency, and regulatory compliance. These committees will collaborate closely with policymakers, industry experts, and local communities to provide oversight and foster trust in AI systems. A notable example comes from the healthcare sector, where similar governance frameworks have been successfully implemented. For instance, the *Clinical AI Governance Committee* at the UK National Health Service (NHS) was established to oversee the ethical deployment of AI tools in patient care. This committee has been instrumental in ensuring that AI systems used in diagnosis and treatment comply with ethical standards, safeguard patient data, and remain transparent in their decision-making processes (NHS England, 2019). Similarly, in the financial sector, AI ethics committees have overseen the responsible use of AI for fraud detection, ensuring compliance with data privacy regulations (Gersh, 2020). These examples show the importance of governance structures in promoting ethical AI practices and mitigating risks across various sectors. These governance structures fostered transparency, facilitated ongoing dialogue, and enabled course corrections when necessary.
- Promoting transparency and accountability in AI decision-making processes: The network recognized the importance of transparency and accountability in AI decision-making processes, particularly in the agricultural sector where decisions can have significant impacts on livelihoods and food security. The AI4AFS team implemented measures to ensure transparency, such as documenting data sources, model architectures, and decision-making processes. Additionally, developing mechanisms for explaining AI-generated recommendations and predictions to stakeholders, will enable them to understand the reasoning behind decisions and provide opportunities for feedback and course correction.

#### 4.1.5 Promoting gender equality and addressing gender disparities

Gender disparities in access to resources, decision-making, and technology adoption continue to persist in many agricultural communities across Africa. This was part of the lessons learned from the project implementation. The team recognized clear strategies to promote gender equality and address these disparities:

- Conducting gender-sensitive needs assessments and stakeholder consultations: Recognizing the unique challenges and barriers faced by women in agriculture, grantees conducted comprehensive gender-sensitive needs assessments and stakeholder consultations. These assessments aimed to understand the specific needs, preferences, and constraints of women farmers, as well as their roles, responsibilities, and decision-making power within households and communities. The gender-disaggregated data and insights, help tailor their AI solutions to address the specific needs and priorities of women farmers.
- Ensuring equal representation and participation of women in project activities: The project team actively sought to ensure equal representation and participation of women in all project activities, from design and development to implementation and evaluation. This involved targeted outreach efforts, capacity-building initiatives, and the creation of inclusive spaces that encouraged women’s voices and perspectives to be heard. When there is a sense of ownership and agency among women stakeholders; this will increase the likelihood of successful technology adoption and sustainable impact.
- Developing gender-responsive AI solutions and user interfaces: There is need for continuous promotion of gender equality by developing gender-responsive AI solutions and user interfaces. This includes incorporating features and functionalities that address the specific needs and preferences of women farmers, such as localized language support, intuitive visual interfaces, and tailored advisory services. Additionally, there is need to also ensure that the data used for training AI models is representative and free from gender biases, reducing the risk of perpetuating existing disparities or perpetuating harmful stereotypes.
- Providing targeted training and capacity-building programs for women farmers and entrepreneurs: Recognizing the digital divide and potential barriers to technology adoption faced by women, grantees implemented targeted training and capacity-building programs. These programs aimed to enhance digital literacy, provide hands-on training in using AI-powered tools and applications, and equip women with entrepreneurial skills to leverage AI solutions for income-generating activities. By investing in women’s capacity building, grantees sought to empower them as active participants and beneficiaries of AI-driven agricultural innovations.

### 4.2 Scaling up responsible AI innovation in agriculture

While the AI4AFS Innovation Research Network has demonstrated the potential of responsible AI solutions in addressing agricultural challenges, scaling up these innovations requires a concerted effort. This section explores policy recommendations, capacity-building strategies, sustainable funding models, and approaches to promote responsible AI adoption and localized impacts across the region.

#### 4.2.1 Policy recommendations for fostering an inclusive technology ecosystem

Policy recommendations for fostering an inclusive technology ecosystem To scale up responsible AI innovation in agriculture and foster an inclusive technology ecosystem, policymakers and stakeholders should consider the following recommendations:

- Developing national and regional AI strategies for agriculture and food systems: Governments should prioritize the development of comprehensive national and regional AI strategies tailored for the agricultural sector. These strategies should outline clear goals, action plans, and investment frameworks to promote responsible AI adoption, address digital infrastructure gaps, and foster capacity-building initiatives. Collaboration among various stakeholders, including policymakers, researchers, civil society organizations, and the private sector, is crucial in formulating these strategies. By establishing a cohesive vision and roadmap, governments can effectively guide the development, deployment, and governance of AI technologies in agriculture, ensuring alignment with local priorities and addressing potential risks and challenges.
- Establishing regulatory frameworks for AI governance and data protection: As AI technologies become more pervasive in agriculture, it is essential to establish robust regulatory frameworks that ensure ethical and responsible development, deployment, and governance of AI systems. These frameworks should address issues such as data privacy, algorithmic bias, transparency, and accountability. Clear guidelines and standards should be developed to govern data collection, storage, and usage practices, particularly when dealing with sensitive agricultural and personal data. Additionally, mechanisms for AI risk assessment, auditing, and monitoring should be implemented to mitigate potential adverse impacts and promote trust in AI systems among stakeholders, including farmers and local communities.
- Investing in digital infrastructure and capacity-building programs: Inadequate digital infrastructure remains a significant barrier to AI adoption in many agricultural regions of Sub-Saharan Africa. Policymakers should prioritize investments in expanding reliable internet connectivity, establishing computing facilities, and developing data storage and management systems. Additionally, comprehensive capacity-building programs should be developed to equip various stakeholders, such as farmers, extension workers, researchers, and policymakers, with the necessary skills and knowledge to effectively utilize and benefit from AI technologies in agriculture. These programs should incorporate hands-on training, knowledge-sharing platforms, and collaboration with educational institutions and research centers.
- Promoting public-private partnerships and multi-stakeholder collaborations: Scaling up responsible AI innovation in agriculture requires concerted efforts from multiple actors, including governments, research institutions, civil society organizations, and private sector companies. Policymakers should foster an enabling environment that encourages public-private partnerships and multi-stakeholder collaborations. These partnerships can leverage the expertise, resources, and capabilities of different stakeholders, facilitating knowledge exchange, joint research and development initiatives, and the co-creation of context-specific AI solutions. By bringing together diverse perspectives and expertise, these collaborations can address complex challenges, promote inclusive participation, and ensure that AI solutions are aligned with local needs and priorities.

#### 4.2.2 Strategies for capacity building and knowledge transfer

Capacity building and knowledge transfer are essential for ensuring the sustained adoption and localized impact of AI solutions in agriculture. Potential strategies include:

- Establishing AI research and innovation hubs in agricultural universities and institutions: Dedicated AI research and innovation hubs should be established within agricultural universities and research institutions across Sub-Saharan Africa. These hubs would serve as centers of excellence, driving cutting-edge research, development, and testing of AI applications for agriculture. By fostering collaborations between academia, industry, and local communities, these hubs can facilitate knowledge exchange, promote interdisciplinary approaches, and facilitate the co-creation of context-specific AI solutions. Additionally, these hubs can offer specialized training programs, workshops, and seminars to build the capacity of students, researchers, and professionals in AI for agriculture.
- Developing tailored training programs for various stakeholders: Comprehensive training programs tailored to the needs of different stakeholders, such as farmers, extension workers, policymakers, and agribusiness professionals, should be developed and implemented. These programs should cover a range of topics, including AI fundamentals, data management, responsible AI principles, and practical applications in agriculture. Hands-on training sessions, demonstrations, and case studies should be incorporated to facilitate knowledge transfer and skill development. Additionally, these programs should be designed to be accessible and inclusive, catering to diverse educational backgrounds and addressing potential language and cultural barriers.
- Fostering knowledge exchange networks and communities of practice: Establishing knowledge exchange networks and communities of practice can facilitate the sharing of best practices, lessons learned, and innovative approaches among stakeholders across different regions and contexts. These networks can leverage digital platforms, social media, and other communication channels to promote collaboration, peer-to-peer learning, and the dissemination of success stories and case studies. Furthermore, regular events, such as conferences, workshops, and webinars, can bring together practitioners, researchers, and policymakers to discuss emerging trends, challenges, and opportunities in AI for agriculture.
- Leveraging digital platforms and e-learning resources for dissemination: The widespread adoption of AI solutions in agriculture can be accelerated by leveraging digital platforms and e-learning resources for knowledge dissemination. Online portals, mobile applications, and interactive multimedia resources can provide accessible and scalable training materials, tutorials, and instructional guides. These resources should be designed to be user-friendly, localized, and tailored to the specific needs and contexts of different stakeholder groups. Additionally, integrating AI-powered features, such as personalized learning paths, virtual assistants, and interactive simulations, can enhance the effectiveness and engagement of these e-learning resources.

#### 4.2.3 Developing sustainable funding models and partnerships

Scaling up responsible AI innovation in agriculture requires sustainable funding models and strategic partnerships. Grantees and stakeholders can explore the following avenues:

- Public-private partnerships and co-investment models: Governments and policymakers should actively promote public-private partnerships (PPPs) and co-investment models to support the development and deployment of AI solutions in agriculture. These partnerships can leverage the complementary strengths of public and private sector entities, combining public funding, research expertise, and policy support with private sector innovation, technological capabilities, and market insights. Co-investment models can take various forms, such as cost-sharing agreements, joint venture initiatives, or collaborative research and development programs. By aligning interests and sharing risks and rewards, PPPs can accelerate the development of AI solutions tailored to local agricultural contexts, ensuring long-term sustainability and scalability.
- Crowdfunding and impact investment opportunities: Crowdfunding platforms and impact investment opportunities can provide alternative funding sources for AI initiatives in agriculture. Crowdfunding campaigns can tap into the collective support of individuals, communities, and organizations passionate about promoting sustainable and inclusive agricultural practices. These campaigns can raise funds for specific AI projects, enabling grassroots innovation and community-driven solutions. Additionally, impact investors, including venture capitalists, angel investors, and social impact funds, can play a crucial role in financing AI startups and enterprises focused on delivering positive social and environmental impacts in agriculture.
- Collaborative grant programs and research consortia: Stakeholders should actively pursue collaborative grant programs and research consortia that bring together diverse partners, including academic institutions, research centers, non-governmental organizations (NGOs), and private companies. These collaborative initiatives can leverage pooled resources, expertise, and complementary capabilities to tackle complex challenges in AI for agriculture. International development agencies, philanthropic organizations, and government agencies can provide funding opportunities and support for such collaborative programs, fostering cross-border and cross-sectoral partnerships that drive innovation and knowledge exchange.
- Engaging with international development organizations and donors: International development organizations, such as the World Bank, United Nations agencies, and bilateral donor agencies, can play a vital role in supporting the adoption of responsible AI in agriculture. These organizations can provide funding, technical assistance, and capacity-building support to developing countries, enabling them to establish the necessary infrastructure, regulatory frameworks, and human capital for AI innovation. Additionally, these organizations can facilitate knowledge sharing, best practice dissemination, and cross-regional collaborations, fostering a global ecosystem that promotes inclusive and sustainable AI adoption in agriculture.

#### 4.2.4 Promoting responsible AI adoption and localized impacts

To ensure responsible AI adoption and localized impacts in agriculture, stakeholders should:

- Prioritize community engagement and co-creation processes: Effective community engagement and co-creation processes are crucial for developing AI solutions that align with local needs, values, and contexts. Stakeholders should prioritize participatory approaches that actively involve farmers, indigenous communities, and local organizations throughout the entire AI development and deployment lifecycle. This includes conducting needs assessments, design workshops, and iterative testing to understand local challenges, incorporate traditional knowledge, and ensure that AI solutions are culturally appropriate and relevant. By fostering a sense of ownership and trust among local communities, these co-creation processes can increase the likelihood of successful adoption and sustained impact.
- Develop context-specific and culturally-appropriate AI solutions: AI solutions should be tailored to the unique agricultural contexts, environmental conditions, and cultural practices of local communities. This may involve adapting algorithms and models to account for regional variations in crop varieties, soil types, climatic patterns, and farming practices. Additionally, user interfaces and decision support systems should be designed with consideration for local languages, literacy levels, and communication preferences. Incorporating traditional knowledge systems and integrating AI with existing agricultural practices can enhance the relevance and acceptance of these solutions among end-users.
- Implement robust monitoring and evaluation frameworks: Robust monitoring and evaluation frameworks are essential for assessing the impact, efficacy, and potential unintended consequences of AI solutions in agriculture. These frameworks should incorporate a range of indicators, including agricultural productivity, environmental sustainability, socioeconomic outcomes, and ethical considerations. Regular data collection, analysis, and stakeholder feedback should inform iterative improvements and adaptations to ensure that AI solutions continue to meet the evolving needs of local communities. Additionally, these frameworks can foster accountability, transparency, and trust in AI systems by providing evidence-based insights and enabling course corrections when necessary.
- Foster cross-sectoral collaborations and knowledge sharing: Promoting responsible AI adoption and localized impacts requires collaboration among diverse stakeholders, including policymakers, researchers, civil society organizations, private sector companies, and local communities. Cross-sectoral collaborations can facilitate knowledge sharing, best practice dissemination, and the identification of synergies and complementary approaches. Establishing platforms for dialogue, such as regional forums, conferences, and online communities of practice, can foster exchange of experiences, lessons learned, and innovative solutions. Furthermore, these collaborations can support the development of harmonized standards, guidelines, and regulatory frameworks, enabling consistent and responsible AI adoption across regions and sectors.

#### 4.2.5 Key insights and future research direction

The study highlights the importance of collaboration among stakeholders in the research and innovation ecosystem. The integration of responsible AI innovations in African agriculture, as demonstrated through the AI4AFS case studies, offers substantial promise for improving productivity, resilience, and sustainability in agriculture. AI-driven tools for pest and disease detection, crop monitoring, and soil health assessment have proven effective in increasing yield and minimizing losses. For example, AI-based pest monitoring and real-time disease detection applications have reduced pest-induced crop losses by as the women benefiting from the intervention showed that that have increased their number of hectare of farming from 1 or 2 hectares to 4–5 hectares, with farmers reporting yield increases. Moreover, AI-enhanced resource allocation systems have led to more efficient water and fertilizer use, optimizing resource inputs by as much as 25% as reported by farmers in Nigeria, Ghana, and Kenya.

However, scaling these innovations requires addressing broader infrastructural and social factors. AI applications in agriculture need robust digital infrastructure and user training to sustain long-term adoption and usability, especially in remote areas with limited internet access. Community-based participatory models, as exemplified in AI4AFS Network’s intervention, enhance adoption and foster inclusivity by integrating local knowledge and supporting marginalized groups, notably women, in technology development and application, which is alluded by with Foster et al. (2023). Through such community-centered approaches, responsible AI can offer globally applicable models for improving agricultural resilience, contributing meaningfully to global food security and climate adaptation efforts. The study suggests that further exploration into AI for sustainable waste management, climate resilience, and biofuel advancements could yield new insights (Diana et al., 2022).

## 5 Conclusion and recommendations

### 5.1 Conclusion

This study underscores the transformative potential of responsible and inclusive AI innovations in addressing critical agricultural challenges in Sub-Saharan Africa, particularly through the insights gained from the Artificial Intelligence for Agriculture and Food Systems (AI4AFS) network. Drawing from the case studies on crop health monitoring, pest detection, and weather forecasting, our findings confirm that AI-driven tools can significantly improve productivity and sustainability when aligned with local contexts and ethical standards. Furthermore, these innovations align with several Sustainable Development Goals (SDGs), notably by enhancing food security (SDG 2), reducing poverty (SDG 1), and supporting gender equality (SDG 5) through targeted solutions that prioritize smallholder farmers, women, and marginalized communities. The results from the research affirms the hypothesis that AI applications tailored to local and gender specific needs significantly impact productivity, with broad implications for agriculture internationally. Our research highlights that, beyond productivity gains, AI applications offer essential capabilities for climate adaptation and resource efficiency, fostering resilience against environmental pressures (SDG 13). It is worthy to note that the integration of AI technologies in agriculture requires supportive infrastructure, capacity-building, and policy frameworks to ensure sustained, responsible, and locally relevant adoption. Thus, this study contributes to a growing body of evidence on the positive impact of AI in agriculture and calls for further research into replicable, inclusive AI models that extend the benefits beyond African contexts, informing global agricultural policies and practices.

### 5.2 Recommendations for future research

1. Expanding AI Applications Beyond Current Focus Areas: Future research should explore the application of AI in other critical areas of agriculture, such as water resource management, climate change adaptation, and supply chain optimization. This expansion could involve developing AI tools that address emerging challenges like drought prediction, efficient water use, and post-harvest loss reduction. This area of research would open up new avenues for junior researchers to specialize in niche aspects of AI in agriculture, providing opportunities for cross-disciplinary collaboration and innovation. Mentorship programs could be established where experienced researchers guide juniors in these emerging fields, fostering a new generation of experts.
2. Investigation of AI’s Socioeconomic Impacts on Smallholder Farmers: Conduct in-depth studies on the socioeconomic impacts of AI adoption on smallholder farmers, particularly focusing on how AI can empower marginalized groups, including women and youth, and reduce inequality in access to resources and decision-making. This research area is rich in qualitative and quantitative opportunities, allowing junior researchers to engage in community-based research, data collection, and analysis. The findings could be used to develop training modules that junior researchers can use to understand the social dimensions of technology adoption, equipping them with skills in both technical and social research methodologies.
3. Longitudinal Studies on AI Integration and Sustainability: Future research should include longitudinal studies that track the long-term sustainability and effectiveness of AI interventions in agriculture. These studies should assess the durability of AI solutions, their adaptability to changing conditions, and their long-term impacts on agricultural productivity and environmental sustainability. Engaging junior researchers in longitudinal studies will provide them with valuable experience in conducting extended research projects, including data management, long-term monitoring, and impact evaluation. These skills are critical for building a career in research and will enhance their ability to contribute to large-scale projects and secure research funding.

## Data Availability

Publicly available datasets were analyzed in this study. This data can be found here: https://atpsice.org/ai4afs/.
